# Evaluation of the NOD/SCID xenograft model for glucocorticoid-regulated gene expression in childhood B-cell precursor acute lymphoblastic leukemia

**DOI:** 10.1186/1471-2164-12-565

**Published:** 2011-11-17

**Authors:** Vivek A Bhadri, Mark J Cowley, Warren Kaplan, Toby N Trahair, Richard B Lock

**Affiliations:** 1 Children's Cancer Institute Australia for Medical Research, Lowy Cancer Research Centre, University of New South Wales, Randwick, NSW 2031, Australia; 2 Peter Wills Bioinformatics Centre, Garvan Institute of Medical Research, Darlinghurst, NSW 2010, Australia; 3 Centre for Children's Cancer and Blood Disorders, Sydney Children's Hospital, Randwick, NSW 2031, Australia

## Abstract

**Background:**

Glucocorticoids such as prednisolone and dexamethasone are critical drugs used in multi-agent chemotherapy protocols used to treat acute lymphoblastic leukemia (ALL), and response to glucocorticoids is highly predictive of outcome. The NOD/SCID xenograft mouse model of ALL is a clinically relevant model in which the mice develop a systemic leukemia which retains the fundamental biological characteristics of the original disease. Here we report a study evaluating the NOD/SCID xenograft mouse model to investigate glucocorticoid-induced gene expression. Cells from a glucocorticoid-sensitive xenograft derived from a child with B-cell precursor ALL were inoculated into NOD/SCID mice. When highly engrafted the mice were randomized into groups of 4 to receive dexamethasone 15 mg/kg by intraperitoneal injection or vehicle control. Leukemia cells were harvested from mice spleens at 0, 8, 24 or 48 hours thereafter, and gene expression analyzed on Illumina WG-6_V3 chips, comparing all groups to time 0 hours.

**Results:**

The 8 hour dexamethasone-treated timepoint had the highest number of significantly differentially expressed genes, with fewer observed at the 24 and 48 hour timepoints, and with minimal changes seen across the time-matched controls. When compared to publicly available datasets of glucocorticoid-induced gene expression from an *in vitro *cell line study and from an *in vivo *study of patients with ALL, at the level of pathways, expression changes in the 8 hour xenograft samples showed a similar response to patients treated with glucocorticoids. Replicate analysis revealed that at the 8 hour timepoint, a dataset with high signal and differential expression, using data from 3 replicates instead of 4 resulted in excellent recovery scores of > 0.9. However at other timepoints with less signal very poor recovery scores were obtained with 3 replicates.

**Conclusions:**

The NOD/SCID xenograft mouse model provides a reproducible experimental system in which to investigate clinically-relevant mechanisms of drug-induced gene regulation in ALL; the 8 hour timepoint provides the highest number of significantly differentially expressed genes; time-matched controls are redundant and excellent recovery scores can be obtained with 3 replicates.

## Background

Glucocorticoids such as prednisolone and dexamethasone are critical components of multi-agent chemotherapy protocols used in the treatment of acute lymphoblastic leukemia (ALL) [1] due to their ability to induce apoptosis in lymphoid cells. Despite their use for over 50 years their mechanism of action is not completely understood. Glucocorticoids are steroid hormones that act on target cells through interaction with a specific glucocorticoid receptor (GR) [2]. The GR is held in a cytosolic complex by a number of co-chaperone molecules including HSP-90 and HSP-70 [3], and on ligand binding dissociates from the co-chaperone complex, dimerizes and is transported to the nucleus where it binds to palindromic DNA sequences known as glucocorticoid response elements (GREs) found in the promoter regions of target genes [4]. This leads to the activation of transcription of primary target genes, repression of transcription through interaction with negative GREs [5] or of gene activation through transcription factors such as AP-1 and NF-ΚB [6]. In lymphoid cells, this results in repression of cell cycle progression through cyclin-D3 and C-MYC [7], and cell death through the activation of apoptosis. Glucocorticoids also induce other non-apoptotic mechanisms of programmed cell death including autophagy [8] and mediate a number of pathways involved in the metabolism of carbohydrates, lipids and proteins.

A number of studies have published microarray data of glucocorticoid-induced genes in lymphoid cells, but comparison of the data is complicated by technical differences in platform and chip type. Previous studies of glucocorticoid-induced genes in ALL have been carried out using *in vitro *cell-line models [9-15] and patient-derived cells, both *in vivo *[16] and *in vitro *[17]. Cell lines are extensively used in the study of ALL but in the process of immortalization acquire multiple genetic defects, particularly in the p53 pathway [18], and mechanisms demonstrated in cell lines are often not replicated in more clinically relevant models. Primary patient cells have a finite supply and rarely survive *ex vivo *for more than a few days. The non-obese diabetic/severe combined immunodeficient (NOD/SCID) xenograft mouse model is widely used to study ALL. In this model, human leukemia cells obtained from diagnostic bone marrow biopsies are inoculated into NOD/SCID mice, and on engraftment establish a systemic leukemia which retains the fundamental biological characteristics of the original disease [19]. It has also been shown that the *in vivo *responses to chemotherapeutic agents, including dexamethasone, correlates with patient outcome [20], and thus the NOD/SCID xenograft mouse model provides a stable, reproducible and clinically relevant model with which to study ALL. Here we report the first study investigating glucocorticoid-induced gene expression in ALL using the NOD/SCID xenograft model, the optimal experimental design, and a comparison of our microarray data to publicly available datasets of glucocorticoid-induced genes in other experimental models.

## Methods

### NOD/SCID xenograft mouse model

All experimental studies were approved by the Human Research Ethics Committee and the Animal Care and Ethics Committee of the University of New South Wales. ALL-3, a glucocorticoid-sensitive xenograft derived from a 12 year old girl with mixed lineage leukemia (MLL)-rearranged BCP-ALL, was chosen for this study. Although MLL-rearranged ALL is associated with a poor prednisolone response and an inferior outcome [21], this patient is currently a long-term survivor. ALL-3 demonstrates *in vitro *glucocorticoid sensitivity, with an IC_50 _of 9.4 nM on exposure to dexamethasone. In the *in vivo *NOD/SCID xenograft mouse model, ALL-3 is highly responsive to 4 weeks of treatment with single agent dexamethasone, with rapid clearance of leukemic blasts from the peripheral blood and recurrence of leukemia delayed by 63.4 days compared to vehicle-treated controls [20].

Cells from ALL-3 were inoculated by tail-vein injection into 28 NOD/SCID mice. The mice were bled weekly and the samples stained with fluorescein isothiocyanate (FITC)-conjugated anti-murine CD45 and allophycocyanin (APC)-conjugated anti-human CD45 (BioLegend, San Diego, CA). Following lysis of erythrocytes with FACS lysing solution (BD Biosciences, San Jose, CA), samples were analyzed by multiparametric flow cytometry on a FACSCanto cytometer (BD Biosciences, San Jose, CA). Engraftment was calculated as the proportion of human versus total CD45^+ ^cells.

When high level (> 70%) engraftment was achieved in the peripheral blood, between 8 and 10 weeks post-transplantation, the mice were randomized and split into groups of 4 to receive either dexamethasone 15 mg/kg (Sigma-Aldrich, St Louis, MO) or vehicle control by intraperitoneal injection. Mice were culled by CO_2 _asphyxiation at 0 hours (pre-treatment, group 1), 8 hours (groups 2 and 3), 24 hours (groups 4 and 5) or 48 hours (groups 6 and 7) following treatment. The mice in groups 6 and 7 received a second dose of dexamethasone or vehicle control at 24 hours. Two mice succumbed early to thymoma, a well-recognized complication in NOD/SCID mice, resulting in 3 mice in each of groups 6 and 7. Cell suspensions of spleens were prepared and mononuclear cells enriched and purified to > 97% human by density gradient centrifugation using LymphoPrep (Axis-Shield, Norway), and cell viability assessed by trypan blue exclusion. RNA was extracted using the RNeasy mini kit (Qiagen, Hilden, Germany) and the RNA integrity verified (Agilent Bioanalyzer, Santa Clara, CA). The RNA was amplified using the Illumina TotalPrep RNA amplification kit (Ambion, Austin, TX) and hybridized onto Illumina WG-6_V3 chips (Illumina, San Diego, CA). The chips were scanned on the Illumina Bead Array Reader (Illumina, San Diego, CA) and gene expression analyzed. The data have been deposited in NCBI's Gene Expression Omnibus [22] and are accessible through GEO Series accession number GSE30392 http://www.ncbi.nlm.nih.gov/geo/query/acc.cgi?acc=GSE30392.

### Gene expression and functional analysis

The sample probe profiles with no normalization or background correction were exported from BeadStudio (version 3.0.14, Illumina, San Diego, CA). The data were pre-processed using variance stabilizing transformation [23] and robust spline normalization in lumi [24] which takes advantage of each probe being represented by > 25 beads. Differential gene expression was determined using limma [25] by comparing all treated groups to time 0 hours, with the positive False Discovery Rate correction for multiple testing [26]. Complete linkage hierarchical clustering using Euclidian distance was used to compare groups to each other. Functional analysis was performed using gene set enrichment analysis (GSEA) version 2.04 [27], comparing the limma moderated t-statistic for each probe in a pre-ranked file, against the c2_all collection of gene sets from the Molecular Signatures Database [27] version 2.5 with 1000 permutations. The similarity of the top 100 up- and down-regulated genesets was assessed using meta-GSEA (Cowley *et al*, manuscript in preparation).

### Comparison of models

The molecular response to glucocorticoids in xenografts was compared to publicly available microarray data [13,16] using parametric analysis of gene set enrichment [28] implemented in the PGSEA package (version 1.20.1, Furge and Dykema) from the Bioconductor project [29], with some modifications to the algorithm to assess significance of the genes that are in the geneset *and represented on the microarray*, and to allow more control over control sample specification (available upon request). Expression levels of each gene in each sample were converted to expression ratios relative to patient matched controls before glucocorticoid treatment (Schmidt *et al*), time-matched controls (Rainer *et al*), or time 0 hours (xenografts). Within each dataset, these gene-level ratios were summarized into geneset-level Z-scores, using PGSEA with genesets from the c2_all collection [27]. The Z-scores from each sample from the 3 studies were combined and then compared by hierarchical clustering of the top 100 gene sets demonstrating the greatest variance across the combined studies.

### Replicate analysis

The stability of results when reducing the number of replicates was assessed using the Recovery Score method [30] from the GeneSelector package (version 1.4.0) of the Bioconductor project [29].

## Results and Discussion

**Figure 1:**
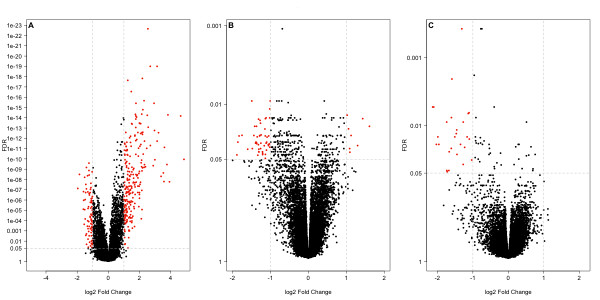
**Volcano plots of significantly differentially expressed genes following treatment with dexamethasone at 8 hours (A), 24 hours (B), 48 hours (C)**. Significance was defined as log2 Fold Change > 1 or < -1 with False Discovery Rate (FDR) < 0.05. Each dot represents a single gene, and significant genes indicated by red dots.

It has been demonstrated that changes in gene expression can be detected as early as 6 hours after treatment of ALL with glucocorticoids *in vivo *[16] and *in vitro *[11], although earlier timepoints show few, if any, significantly differentially expressed genes [17]. In this study the 8 hour dexamethasone-treated timepoint demonstrated the highest number of differentially expressed genes compared to baseline control, with far fewer observed at the 24 and 48 hour dexamethasone-treated timepoints (Tables 1 and 2, and Figure 1). Whilst a similar proportion of up- and down-regulated genes were identified at the 8 hour dexamethasone-treated timepoint (1158 vs 1072 respectively, FDR < 0.05), of those with large fold changes (FC > 2 or FC < 0.5, red dots in Figure 1A), 75% were up regulated (199 vs 65 respectively), consistent with the predominant role of glucocorticoids as transcriptional activators. The large numbers of statistically differentially expressed genes (FDR < 0.05) with small fold changes (0.5 < FC < 2) are indicative of both small measurement error across replicates, and thus the high reproducibility of the xenograft model, and good experimental power resulting from using 4 replicates. There was minimal significant differential gene expression across the time-matched controls (Tables 1 and 2). This demonstrates that in the xenograft mouse model, the 8 hour timepoint provides the greatest information, and that these changes are not sustained over later timepoints. The handling of the mice and intraperitoneal vehicle control injections had minimal effect on gene expression, and thus time-matched controls are redundant.

**Table 1:** Number of differentially expressed genes by False Discovery Rate (FDR), compared to time 0 hours.

Timepoint (hours)	FDR < 0.25		FDR < 0.1		FDR < 0.05	
	+	-	+	-	+	-
**Dex 8**	2313	2434	1470	1423	1158	1072
**Dex 24**	970	1087	273	421	75	195
**Dex 48**	321	327	41	95	12	44
**Con 8**	0	0	0	0	0	0
**Con 24**	0	1	0	1	0	1
**Con 48**	0	1	0	1	0	1

**+ **upregulated; **- **downregulated; Dex, dexamethasone-treated; and Con, control

**Table 2:** Number of differentially expressed genes by Fold Change (FC), compared to time 0 hours.

Timepoint (hours)	FC > 1.5		FC > 2		FC > 4	
	+	-	+	-	+	-
**Dex 8**	501	429	201	68	38	0
**Dex 24**	137	341	15	90	0	0
**Dex 48**	79	234	5	69	0	3
**Con 8**	1	37	1	2	0	0
**Con 24**	1	5	0	0	0	0
**Con 48**	7	34	0	2	0	0

**+ **upregulated; **- **downregulated; Dex, dexamethasone-treated; and Con, control

At the 8 hour timepoint, there were 173 genes upregulated with a t-statistic (the ratio of fold change to standard error) > 10 and 25 genes downregulated with a t-statistic < -10 (corresponding to P < 1.74 × 10^-9 ^and FDR < 2.95 × 10^-7^, table 3). None of these genes had sustained expression changes at 24 or 48 hours, and although this could potentially reflect the early death of sensitive cells, there was no significant difference in the total number of cells harvested from the spleens at any timepoint compared to the time-matched controls (data not shown), and all harvests had a cell viability of ≥ 96%.

**Table 3:** Genes regulated 8 hours following dexamethasone treatment.

ProbeSet ID	Gene	t	P	FDR	Definition
**Upregulated**					
ILMN_5080450	ZBTB16	83.77	< 2.2E-16	< 2.2E-16	zinc finger and BTB domain containing 16
ILMN_3800088	MMP7	53.22	< 2.2E-16	< 2.2E-16	matrix metallopeptidase 7
ILMN_1770593	CH25H	53.14	< 2.2E-16	< 2.2E-16	cholesterol 25-hydroxylase
ILMN_6560328	C6orf85	44.60	< 2.2E-16	< 2.2E-16	chromosome 6 open reading frame 85
ILMN_7570561	TSC22D3	39.16	< 2.2E-16	< 2.2E-16	TSC22 domain family, member 3
ILMN_580187	PDE8B	33.88	< 2.2E-16	3.90E-16	phosphodiesterase 8B
ILMN_5130066	C8orf61	33.82	< 2.2E-16	3.90E-16	chromosome 8 open reading frame 61
ILMN_4120431	TMEM100	31.38	< 2.2E-16	1.64E-15	transmembrane protein 100
ILMN_650553	BIN1	29.76	< 2.2E-16	4.43E-15	bridging integrator 1
ILMN_1400373	SLA	29.57	< 2.2E-16	4.63E-15	Src-like-adaptor
ILMN_6330593	PTHR1	29.28	< 2.2E-16	5.22E-15	parathyroid hormone receptor 1
ILMN_6110037	LILRA3	29.04	< 2.2E-16	5.75E-15	leukocyte immunoglobulin-like receptor subfamily A, member 3
ILMN_4150477	LOXL4	28.67	< 2.2E-16	6.66E-15	lysyl oxidase-like 4
ILMN_2680079	OGFRL1	28.65	< 2.2E-16	6.66E-15	opioid growth factor receptor-like 1
ILMN_4210411	NDRG2	28.20	< 2.2E-16	8.62E-15	NDRG family member 2
ILMN_3780093	LILRA1	27.86	< 2.2E-16	1.05E-14	leukocyte immunoglobulin-like receptor subfamily A, member 1
ILMN_240441	IL1R2	27.46	< 2.2E-16	1.33E-14	interleukin 1 receptor, type II
ILMN_4730315	MERTK	26.14	< 2.2E-16	3.31E-14	c-mer proto-oncogene tyrosine kinase
ILMN_3800538	ACPL2	25.90	< 2.2E-16	3.72E-14	acid phosphatase-like 2
ILMN_6860392	UGT2B17	25.83	< 2.2E-16	3.72E-14	UDP glucuronosyltransferase 2 family, polypeptide B17
ILMN_4730541	SLC44A1	25.82	< 2.2E-16	3.72E-14	solute carrier family 44, member 1
ILMN_4860546	CTHRC1	25.64	< 2.2E-16	4.10E-14	collagen triple helix repeat containing 1
ILMN_3460270	ZHX3	24.56	< 2.2E-16	8.79E-14	zinc fingers and homeoboxes 3
ILMN_10639	RASSF4	23.21	< 2.2E-16	2.57E-13	Ras association (RalGDS/AF-6) domain family 4
ILMN_1190064	UGT2B7	23.13	< 2.2E-16	2.67E-13	UDP glucuronosyltransferase 2 family, polypeptide B7
ILMN_6400603	MGC2463	23.06	< 2.2E-16	2.71E-13	poliovirus receptor related immunoglobulin domain containing
ILMN_3450187	IRGM	23.04	< 2.2E-16	2.71E-13	immunity-related GTPase family, M
ILMN_6620528	MT1X	22.95	2.40E-16	2.85E-13	metallothionein 1X
ILMN_1260341	IL13RA1	22.47	3.67E-16	4.13E-13	interleukin 13 receptor, alpha 1
ILMN_2650112	SLC16A2	22.25	4.48E-16	4.91E-13	solute carrier family 16, member 2
ILMN_5570170	PNMT	22.01	5.59E-16	5.95E-13	phenylethanolamine N-methyltransferase
ILMN_870376	C9orf152	21.93	6.02E-16	6.25E-13	chromosome 9 open reading frame 152
ILMN_3190379	TGFBR3	21.52	8.78E-16	8.89E-13	transforming growth factor, beta receptor III
ILMN_1780142	DSCR1	21.08	1.33E-15	1.31E-12	Down syndrome critical region gene 1
ILMN_2640341	FKBP5	20.63	2.05E-15	1.89E-12	FK506 binding protein 5
ILMN_7610136	LOC652626	20.43	2.48E-15	2.23E-12	Leukocyte immunoglobulin-like receptor subfamily B member 2
ILMN_1410609	CORO2A	20.34	2.72E-15	2.35E-12	coronin, actin binding protein, 2A
ILMN_1780088	TBXA2R	20.29	2.84E-15	2.40E-12	thromboxane A2 receptor
ILMN_270431	BAALC	20.23	3.02E-15	2.50E-12	brain and acute leukemia, cytoplasmic
ILMN_6280176	GBE1	20.02	3.72E-15	3.01E-12	glucan (1,4-alpha-), branching enzyme 1
ILMN_6060113	TBX15	19.81	4.62E-15	3.67E-12	T-box 15
ILMN_4890743	IQSEC1	19.71	5.09E-15	3.97E-12	IQ motif and Sec7 domain 1
ILMN_150056	DPEP1	19.65	5.41E-15	4.13E-12	dipeptidase 1
ILMN_2060364	BTNL9	19.26	8.04E-15	5.91E-12	butyrophilin-like 9
ILMN_3830735	UPB1	19.23	8.30E-15	5.91E-12	ureidopropionase, beta
ILMN_5670377	STYK1	19.15	9.09E-15	6.35E-12	serine/threonine/tyrosine kinase 1
ILMN_4390630	STAG3	18.72	1.42E-14	9.39E-12	stromal antigen 3
ILMN_4070048	NPHP4	18.44	1.91E-14	1.25E-11	nephronophthisis 4
ILMN_4220474	C6orf81	18.31	2.16E-14	1.39E-11	chromosome 6 open reading frame 81
ILMN_1470746	PTPN3	18.30	2.23E-14	1.41E-11	protein tyrosine phosphatase, non-receptor type 3
ILMN_5860576	C20orf133	18.25	2.36E-14	1.47E-11	MACRO domain containing 2
ILMN_6020468	PPP1R14A	18.18	2.52E-14	1.55E-11	protein phosphatase 1, regulatory (inhibitor) subunit 14A
ILMN_1400634	MT1M	18.10	2.76E-14	1.64E-11	metallothionein 1M
ILMN_4250315	ITGA9	17.90	3.46E-14	2.03E-11	integrin, alpha 9
ILMN_5080471	MAP3K6	17.40	6.02E-14	3.44E-11	mitogen-activated protein kinase 6
ILMN_5360242	FLJ42461	17.36	6.28E-14	3.53E-11	smoothelin-like 2
ILMN_6620402	NUDT16	17.33	6.50E-14	3.60E-11	nudix (nucleoside diphosphate linked moiety X)-type motif 16
ILMN_3360112	TMEM2	17.26	7.04E-14	3.85E-11	transmembrane protein 2
ILMN_6840743	PER1	17.22	7.41E-14	3.99E-11	period homolog 1
ILMN_4220347	LRRC1	17.12	8.29E-14	4.33E-11	leucine rich repeat containing 1
ILMN_4850592	P2RY14	17.11	8.35E-14	4.33E-11	purinergic receptor P2Y, G-protein coupled, 14
ILMN_6560300	SLC31A2	16.91	1.05E-13	5.39E-11	solute carrier family 31 member 2
ILMN_4060091	DKFZ	16.87	1.11E-13	5.62E-11	DKFZp451A211 protein
ILMN_6770370	LOC92196	16.28	2.23E-13	1.11E-10	death associated protein-like 1
ILMN_580487	IL9R	16.21	2.40E-13	1.18E-10	interleukin 9 receptor
ILMN_1990300	SOCS1	16.18	2.49E-13	1.21E-10	suppressor of cytokine signaling 1
ILMN_5720424	NRP1	16.17	2.54E-13	1.22E-10	neuropilin 1
ILMN_4180427	CIB4	16.11	2.74E-13	1.30E-10	calcium and integrin binding family member 4
ILMN_4180544	ROPN1L	16.08	2.81E-13	1.32E-10	ropporin 1-like
ILMN_4250167	SOX13	16.04	2.95E-13	1.37E-10	SRY (sex determining region Y)-box 13
ILMN_6330170	CHKA	15.81	3.94E-13	1.81E-10	choline kinase alpha, 3
ILMN_4560192	SFXN5	15.62	4.95E-13	2.25E-10	sideroflexin 5
ILMN_2810136	CAPN11	15.56	5.33E-13	2.40E-10	calpain 11
ILMN_2690709	VIPR1	15.38	6.68E-13	2.91E-10	vasoactive intestinal peptide receptor 1
ILMN_630091	NCOA7	15.38	6.69E-13	2.91E-10	nuclear receptor coactivator 7
ILMN_5390730	MGC17330	15.21	8.25E-13	3.55E-10	phosphoinositide-3-kinase interacting protein 1
ILMN_130364	MST150	15.19	8.49E-13	3.62E-10	MSTP150
ILMN_3450241	KIAA0774	14.95	1.16E-12	4.77E-10	KIAA0774
ILMN_2230678	ACACB	14.80	1.41E-12	5.76E-10	acetyl-Coenzyme A carboxylase beta
ILMN_5870307	LOC440359	14.78	1.44E-12	5.83E-10	similar to muscle Y-box protein YB2
ILMN_3840554	SPOCK2	14.76	1.49E-12	5.95E-10	sparc/osteonectin, cwcv and kazal-like domains 2
ILMN_5810600	MAP3K5	14.69	1.63E-12	6.47E-10	mitogen-activated protein kinase 5
ILMN_2360719	IRAK3	14.65	1.71E-12	6.65E-10	interleukin-1 receptor-associated kinase 3
ILMN_1510121	MTSS1	14.64	1.73E-12	6.66E-10	metastasis suppressor 1
ILMN_540671	LILRB2	14.54	1.98E-12	7.41E-10	leukocyte immunoglobulin-like receptor subfamily B, member 2
ILMN_6980377	MTMR15	14.44	2.26E-12	8.39E-10	myotubularin related protein 15
ILMN_6220288	PRDM1	14.43	2.28E-12	8.39E-10	PR domain containing 1, with ZNF domain
ILMN_7330739	NDRG4	14.42	2.30E-12	8.39E-10	NDRG family member 4
ILMN_2600470	WDR60	14.20	3.10E-12	1.12E-09	WD repeat domain 60
ILMN_4050441	SH3MD4	14.16	3.27E-12	1.17E-09	SH3 multiple domains 4
ILMN_6760546	TIPARP	13.89	4.74E-12	1.64E-09	TCDD-inducible poly(ADP-ribose) polymerase
ILMN_2760537	MTE	13.89	4.75E-12	1.64E-09	metallothionein E
ILMN_160019	SORT1	13.79	5.44E-12	1.83E-09	sortilin 1
ILMN_6330132	ISG20	13.60	7.00E-12	2.32E-09	interferon stimulated exonuclease gene 20 kDa
ILMN_1510685	DOK4	13.52	7.86E-12	2.58E-09	docking protein 4
ILMN_1240228	PAG1	13.47	8.50E-12	2.77E-09	phosphoprotein associated glycosphingolipid microdomains 1
ILMN_580592	CPNE8	13.32	1.04E-11	3.31E-09	copine VIII
ILMN_5870301	KIAA0513	13.32	1.05E-11	3.31E-09	KIAA0513
ILMN_20129	CD52	13.32	1.05E-11	3.31E-09	CD52 molecule
ILMN_1820386	PARVB	13.31	1.06E-11	3.31E-09	parvin, beta
ILMN_6200402	MT1A	13.24	1.17E-11	3.64E-09	metallothionein 1A
ILMN_290661	CLN8	13.10	1.43E-11	4.36E-09	ceroid-lipofuscinosis, neuronal 8
ILMN_670082	GNA12	13.08	1.47E-11	4.43E-09	guanine nucleotide binding protein (G protein) alpha 12
ILMN_5570286	TACC2	12.99	1.67E-11	5.00E-09	transforming, acidic coiled-coil containing protein 2
ILMN_3190411	STARD13	12.93	1.81E-11	5.32E-09	START domain containing 13
ILMN_4540138	NGB	12.92	1.85E-11	5.39E-09	neuroglobin
ILMN_2000646	B4GALT4	12.83	2.10E-11	6.07E-09	UDP-galactosyltransferase, polypeptide 4
ILMN_7100731	CYGB	12.81	2.17E-11	6.17E-09	cytoglobin
ILMN_7050113	NTRK1	12.71	2.52E-11	7.09E-09	neurotrophic tyrosine kinase receptor, type 1
ILMN_2490670	GNPTAB	12.66	2.71E-11	7.52E-09	N-acetylglucosamine-1-phosphate transferase, alpha and beta
ILMN_20170	ZNF385	12.48	3.55E-11	9.72E-09	zinc finger protein 385
ILMN_2630687	CHPT1	12.43	3.80E-11	1.02E-08	choline phosphotransferase 1
ILMN_4120215	WASF2	12.43	3.81E-11	1.02E-08	WAS protein family, member 2
ILMN_5260494	TMLHE	12.39	4.06E-11	1.08E-08	trimethyllysine hydroxylase, epsilon
ILMN_5220333	C14orf139	12.31	4.54E-11	1.20E-08	chromosome 14 open reading frame 139
ILMN_3850440	FCER1G	12.12	6.07E-11	1.60E-08	Fc fragment of IgE, receptor for; gamma polypeptide
ILMN_1030008	TGFB3	12.11	6.21E-11	1.63E-08	transforming growth factor, beta 3
ILMN_1450468	MYT1	12.02	7.04E-11	1.81E-08	myelin transcription factor 1
ILMN_7560541	SLC2A5	12.01	7.19E-11	1.83E-08	solute carrier family 2 member 5
ILMN_2030438	GBA2	12.01	7.21E-11	1.83E-08	glucosidase, beta (bile acid) 2
ILMN_6840328	SMAD3	12.00	7.35E-11	1.86E-08	SMAD family member 3
ILMN_3930390	SMAP1L	11.91	8.40E-11	2.11E-08	stromal membrane-associated protein 1-like
ILMN_7570196	TSPAN9	11.90	8.54E-11	2.12E-08	tetraspanin 9
ILMN_6980546	CACNA1I	11.90	8.56E-11	2.12E-08	calcium channel, voltage-dependent, T type, alpha 1I subunit
ILMN_1710364	LCN6	11.89	8.72E-11	2.15E-08	lipocalin 6
ILMN_5360424	RPS6KA2	11.77	1.04E-10	2.54E-08	ribosomal protein S6 kinase, 90 kDa, polypeptide 2
ILMN_5890193	MS4A4A	11.72	1.14E-10	2.75E-08	membrane-spanning 4-domains, subfamily A, member 4
ILMN_3390292	KLF9	11.66	1.24E-10	2.98E-08	Kruppel-like factor 9
ILMN_5720059	GFOD1	11.65	1.26E-10	3.02E-08	glucose-fructose oxidoreductase domain containing 1
ILMN_7650523	TMEM46	11.57	1.43E-10	3.39E-08	transmembrane protein 46
ILMN_5700392	LOC654000	11.46	1.70E-10	3.95E-08	ribosome biogenesis protein BMS1 homolog 2
ILMN_4810348	C1orf188	11.40	1.88E-10	4.33E-08	chromosome 1 open reading frame 188
ILMN_4280180	CHRNA3	11.39	1.91E-10	4.37E-08	cholinergic receptor, nicotinic, alpha 3
ILMN_270458	CRISPLD1	11.37	1.96E-10	4.45E-08	cysteine-rich secretory protein LCCL domain containing 1
ILMN_450615	MT2A	11.37	1.97E-10	4.46E-08	metallothionein 2A
ILMN_20470	GRASP	11.35	2.02E-10	4.51E-08	GRP1-associated scaffold protein
ILMN_3370594	LILRA2	11.35	2.03E-10	4.51E-08	leukocyte immunoglobulin-like receptor subfamily A, member 2
ILMN_5220397	RREB1	11.34	2.05E-10	4.53E-08	ras responsive element binding protein 1
ILMN_1410192	TDRD9	11.34	2.07E-10	4.56E-08	tudor domain containing 9
ILMN_4070259	LOC653133	11.27	2.30E-10	4.99E-08	guanine nucleotide binding protein (G protein) alpha 12
ILMN_5960682	RBPMS2	11.24	2.41E-10	5.21E-08	RNA binding protein with multiple splicing 2
ILMN_1440300	SLC27A3	11.22	2.50E-10	5.37E-08	solute carrier family 27, member 3
ILMN_5050768	LONRF1	11.20	2.58E-10	5.53E-08	LON peptidase N-terminal domain and ring finger 1
ILMN_6270273	KHDRBS3	11.18	2.67E-10	5.68E-08	KH domain, RNA binding, signal transduction associated 3
ILMN_7100603	KCNK3	11.17	2.70E-10	5.72E-08	potassium channel, subfamily K, member 3
ILMN_2320129	CSDA	11.03	3.38E-10	7.08E-08	cold shock domain protein A
ILMN_3930022	LOC644739	10.99	3.63E-10	7.54E-08	Wiskott-Aldrich syndrome protein family member 4
ILMN_7400133	CUGBP2	10.90	4.20E-10	8.63E-08	CUG triplet repeat, RNA binding protein 2
ILMN_3290301	FZD8	10.88	4.33E-10	8.76E-08	frizzled homolog 8
ILMN_7320520	MTUS1	10.88	4.33E-10	8.76E-08	mitochondrial tumor suppressor 1
ILMN_3780053	PALLD	10.82	4.79E-10	9.60E-08	palladin, cytoskeletal associated protein
ILMN_6860162	LOC441019	10.74	5.49E-10	1.09E-07	hypothetical LOC441019
ILMN_5810154	ALOX15B	10.74	5.50E-10	1.09E-07	arachidonate 15-lipoxygenase, type B
ILMN_3930736	CHST3	10.73	5.59E-10	1.09E-07	carbohydrate (chondroitin 6) sulfotransferase 3
ILMN_60470	STX11	10.72	5.68E-10	1.10E-07	syntaxin 11
ILMN_3390484	SERINC2	10.69	5.95E-10	1.15E-07	serine incorporator 2
ILMN_1430647	TAX1BP3	10.61	6.82E-10	1.31E-07	Tax1 (human T-cell leukemia virus type I) binding protein 3
ILMN_5960440	VDR	10.60	6.99E-10	1.34E-07	vitamin D (1,25-dihydroxyvitamin D3) receptor
ILMN_6290735	EPHB3	10.51	8.10E-10	1.53E-07	EPH receptor B3
ILMN_2680372	SH2D4A	10.46	8.78E-10	1.64E-07	SH2 domain containing 4A
ILMN_2480050	SOX7	10.44	9.13E-10	1.69E-07	SRY (sex determining region Y)-box 7
ILMN_130128	LOC285016	10.41	9.61E-10	1.76E-07	hypothetical protein LOC285016
ILMN_4890451	GRAMD3	10.39	9.87E-10	1.80E-07	GRAM domain containing 3
ILMN_770161	C10orf73	10.39	9.92E-10	1.81E-07	chromosome 10 open reading frame 73
ILMN_2450202	KIF3C	10.35	1.05E-09	1.88E-07	kinesin family member 3C
ILMN_6840468	HAL	10.35	1.06E-09	1.89E-07	histidine ammonia-lyase
ILMN_2470070	TBL1X	10.30	1.15E-09	2.04E-07	transducin (beta)-like 1X-linked
ILMN_2320114	KLF13	10.27	1.22E-09	2.15E-07	Kruppel-like factor 13
ILMN_6380112	DIP	10.23	1.31E-09	2.27E-07	death-inducing-protein
ILMN_2470358	IFNGR1	10.22	1.32E-09	2.30E-07	interferon gamma receptor 1
ILMN_4250735	IL27RA	10.07	1.70E-09	2.91E-07	interleukin 27 receptor, alpha
ILMN_1470215	MAP3K8	10.07	1.72E-09	2.91E-07	mitogen-activated protein kinase 8
ILMN_2940373	TACC1	10.06	1.74E-09	2.94E-07	transforming, acidic coiled-coil containing protein 1
**Downregulated**					
ILMN_770538	LYSMD2	-15.49	5.81E-13	2.58E-10	LysM, putative peptidoglycan-binding, domain containing 2
ILMN_7150059	STAMBPL1	-14.61	1.79E-12	6.84E-10	STAM binding protein-like 1
ILMN_5340692	STRBP	-14.56	1.93E-12	7.31E-10	spermatid perinuclear RNA binding protein
ILMN_4210397	GLDC	-14.05	3.80E-12	1.34E-09	glycine dehydrogenase
ILMN_6980327	DKC1	-13.79	5.44E-12	1.83E-09	dyskeratosis congenita 1, dyskerin
ILMN_50086	TCF12	-13.23	1.19E-11	3.69E-09	transcription factor 12
ILMN_4860356	BYSL	-12.81	2.17E-11	6.17E-09	bystin-like
ILMN_4280228	IVNS1ABP	-12.70	2.55E-11	7.12E-09	influenza virus NS1A binding protein
ILMN_1990379	SLFN11	-11.82	9.63E-11	2.36E-08	schlafen family member 11
ILMN_5220338	MPEG1	-11.64	1.27E-10	3.03E-08	macrophage expressed gene 1
ILMN_450168	SFRS7	-11.50	1.60E-10	3.74E-08	splicing factor, arginine/serine-rich 7, 35 kDa
ILMN_3460687	KIAA0690	-11.42	1.81E-10	4.19E-08	ribosomal RNA processing 12 homolog
ILMN_3400360	MAPRE2	-11.36	1.99E-10	4.48E-08	microtubule-associated protein, RP/EB family, member 2
ILMN_4010414	PPFIBP1	-11.12	2.92E-10	6.16E-08	PTPRF interacting protein, binding protein 1 (liprin beta 1)
ILMN_1190139	UGT3A2	-10.99	3.61E-10	7.54E-08	UDP glycosyltransferase 3 family, polypeptide A2
ILMN_4150201	BCL2	-10.93	3.99E-10	8.24E-08	B-cell CLL/lymphoma 2
ILMN_780240	C12orf24	-10.85	4.53E-10	9.13E-08	chromosome 12 open reading frame 24
ILMN_6760167	MARCH3	-10.73	5.60E-10	1.09E-07	membrane-associated ring finger (C3HC4) 3
ILMN_3940615	PUS7	-10.52	7.99E-10	1.52E-07	pseudouridylate synthase 7 homolog
ILMN_20544	GART	-10.41	9.53E-10	1.76E-07	phosphoribosylglycinamide formyltransferase
ILMN_2480326	HSP90B1	-10.36	1.05E-09	1.88E-07	heat shock protein 90 kDa beta (Grp94), member 1
ILMN_5270367	CTSC	-10.25	1.26E-09	2.20E-07	cathepsin C
ILMN_5420095	MYC	-10.21	1.36E-09	2.34E-07	v-myc myelocytomatosis viral oncogene homolog
ILMN_4610180	PIK3C2B	-10.20	1.38E-09	2.37E-07	phosphoinositide-3-kinase, class 2, beta polypeptide
ILMN_6450300	GEMIN4	-10.00	1.95E-09	3.27E-07	gem (nuclear organelle) associated protein 4

t, t-statistic; and FDR, false discovery rate

The most significantly differentially expressed gene at the 8 hour dexamethasone-treated timepoint was *ZBTB16*, a known transcriptional repressor and glucocorticoid response gene, which has been shown to modulate glucocorticoid sensitivity in CEM T-ALL cells [31]. Other known glucocorticoid response genes upregulated included *TSC22D3 *[32] and *SOCS1 *[15], both downstream targets of the glucocorticoid receptor, *FKBP5 *[33], a co-chaperone of the glucocorticoid receptor, and MAP kinases 5, 6 and 8 [34]. Downregulated genes at 8 hours included *BCL-2 *[35] and *C-MYC *[36], both previously described, but also *HSP90B1*, a glucocorticoid receptor co-chaperone and regulator of apoptosis. The only pro-apoptotic gene consistently upregulated across multiple microarray analyses is the BH3-only BCL-2 family member *BIM*, and it has been shown that BIM has a critical role in glucocorticoid sensitivity and resistance [37], although in this current study *BIM *was only induced 1.3 fold. Thus these genes identified are consistent with previous reports of glucocorticoid-induced genes in ALL. Within these experimental systems however there are significant potential differences in glucocorticoid exposure between *in vitro *and *in vivo *models - a crucial one is that cells *in vitro *are continuously exposed to glucocorticoid whereas in *in vivo *models the glucocorticoid is subject to pharmacokinetic and pharmacodynamic changes which more accurately reflect changes in patients.

At the later timepoints, significant differential gene expression was much less marked and predominantly downregulated. At 24 hours 5 genes were upregulated (t-statistic > 6) and 10 genes downregulated (t-statistic < -6, table 4), and at 48 hours 1 gene was upregulated (t-statistic > 6) and 15 genes downregulated (t-statistic < -6, table 5). At 24 hours, upregulated genes included *NFKBIA*, an inhibitor of NF-ΚB, and *TRIM74*, which was sustained at 48 hours, the significance of which is uncertain. Downregulated genes were those involved in cell cycle progression, including *CCNF *at 24 hours, and *CCNF*, *CDC20 *and *AURKA *at 48 hours, consistent with growth arrest.

**Table 4:** Genes regulated 24 hours following dexamethasone treatment.

ProbeSet ID	Gene	t	P	FDR	Definition
**Upregulated**					
ILMN_3930687	FAM112A	6.67	1.32E-06	0.0091	family with sequence similarity 112, member A
ILMN_6620255	TRIM74	6.29	3.06E-06	0.0132	tripartite motif-containing 74
ILMN_4280113	NFKBIA	6.23	3.48E-06	0.0138	nuclear factor kappa B inhibitor, alpha
ILMN_2140136	EMR2	6.10	4.65E-06	0.0149	egf-like containing, mucin-like, hormone receptor-like 2
ILMN_7000397	ANKRD15	6.08	4.91E-06	0.0149	ankyrin repeat domain 15
**Downregulated**					
ILMN_870524	HOXB8	-8.60	2.53E-08	0.0011	homeo box B8
ILMN_4830520	LOC144501	-6.72	1.19E-06	0.0091	hypothetical protein LOC144501
ILMN_6110332	ARHGAP19	-6.70	1.24E-06	0.0091	Rho GTPase activating protein 19
ILMN_2970619	ESPL1	-6.65	1.38E-06	0.0091	extra spindle pole bodies homolog 1
ILMN_3130541	CCNF	-6.64	1.43E-06	0.0091	cyclin F
ILMN_4760577	CENPA	-6.62	1.46E-06	0.0091	centromere protein A
ILMN_4810646	PIF1	-6.54	1.76E-06	0.0095	PIF1 5'-to-3' DNA helicase homolog
ILMN_1070762	PSRC1	-6.40	2.38E-06	0.0114	proline/serine-rich coiled-coil 1
ILMN_4860703	LOC648695	-6.19	3.82E-06	0.0138	retinoblastoma binding protein 4
ILMN_1110538	INCENP	-6.05	5.19E-06	0.0149	inner centromere protein antigens 135/155 kDa

t, t-statistic; and FDR, false discovery rate

**Table 5:** Genes regulated 48 hours following dexamethasone treatment.

ProbeSet ID	Gene	t	P	FDR	Definition
**Upregulated**					
ILMN_6620255	TRIM74	6.30	3.01E-06	0.0089	tripartite motif-containing 74
**Downregulated**					
ILMN_4810646	PIF1	-8.85	1.58E-08	0.0004	PIF1 5'-to-3' DNA helicase homolog
ILMN_870524	HOXB8	-8.66	2.26E-08	0.0004	homeo box B8
ILMN_1450193	LGALS1	-8.57	2.66E-08	0.0004	lectin, galactoside-binding, soluble, 1 (galectin 1)
ILMN_4760577	CENPA	-7.64	1.71E-07	0.0018	centromere protein A
ILMN_4730605	AURKA	-7.47	2.42E-07	0.0021	aurora kinase A
ILMN_1500010	CDC20	-6.84	9.09E-07	0.0053	CDC20 cell division cycle 20 homolog
ILMN_4060064	HMMR	-6.82	9.61E-07	0.0053	hyaluronan-mediated motility receptor
ILMN_2070408	MID1	-6.80	9.97E-07	0.0053	midline 1 (Opitz/BBB syndrome)
ILMN_2070288	MT1E	-6.66	1.36E-06	0.0065	metallothionein 1E
ILMN_1070762	PSRC1	-6.60	1.55E-06	0.0067	proline/serine-rich coiled-coil 1
ILMN_150543	C20orf129	-6.46	2.12E-06	0.0077	chromosome 20 open reading frame 129
ILMN_5870193	FAM64A	-6.45	2.14E-06	0.0077	family with sequence similarity 64, member A
ILMN_2810201	KIF14	-6.34	2.77E-06	0.0089	kinesin family member 14
ILMN_1050195	KIF20A	-6.28	3.11E-06	0.0089	kinesin family member 20A
ILMN_3130541	CCNF	-6.05	5.21E-06	0.0131	cyclin F

t, t-statistic; and FDR, false discovery rate

Functional analysis using GSEA and meta-GSEA on the expression profiles obtained at 8 hours and 24 hours after dexamethasone treatment (additional files 1 and 2), revealed a significant upregulation of metabolic pathways, particularly adipogenesis at 8 hours, and a marked effect on pathways associated with cell cycling and proliferation, particularly downregulation of C-MYC at 8 hours and NF-ΚB at 24 hours, and upregulation of apoptotic pathways at 24 hours. Glucocorticoids are known to have effects on multiple cellular metabolic pathways, including glucose and carbohydrate metabolism, and have pro-adipogenic effects [38]. Suppression of C-MYC is a critical step prior to the initiation of apoptosis by dexamethasone in ALL [39] and suppression of NF-ΚB has been described [40].

To determine whether the molecular response to glucocorticoids in this xenograft model of ALL mimicked the effects seen in either glucocorticoid-treated patients with ALL [16] or cell-line models of ALL [13], we applied parametric gene set enrichment analysis (PGSEA) [28]. Comparing gene expression profiles from multiple experiments is notoriously difficult and typically any true similarities are swamped by technical differences in microarray vendor, normalization strategies and analytical approach. By summarizing genes at the gene set level (such as genes in the same pathway), these technical differences are mitigated, allowing comparison of samples from multiple studies.

**Figure 2:**
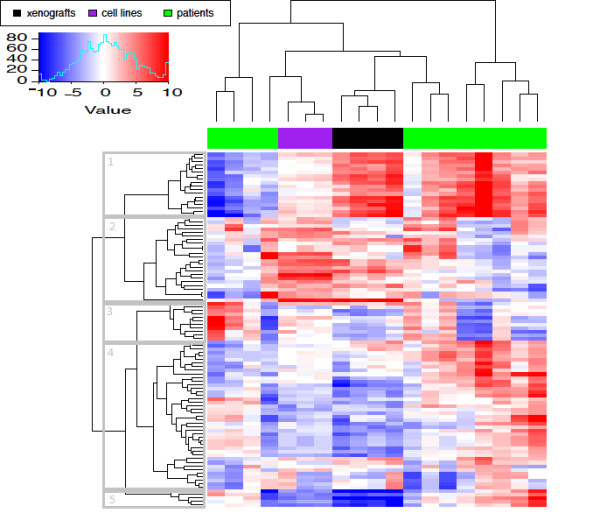
**Parametric GSEA of combined top 100 glucocorticoid-induced gene sets with greatest variance from xenograft, patient and cell line models**. Hierarchical clustering with gene sets in rows, samples in columns (xenografts - black, patient - green, cell line - purple). Each colour of each cell represents the Z-score (see legend). Boxes 1-5 represent defined clusters.

We performed PGSEA on the 6-8 hour samples from the 3 studies, and then two-dimensional hierarchical clustering to identify the relationships between the different ALL models (Figure 2 and annotated in additional file 3). This revealed considerable heterogeneity in the molecular response to glucocorticoids in patients into at least 2, and possibly 4 different groups (green bars, Figure 2), which may represent different modes of response to glucocorticoids in patients. Relative to this inter-patient heterogeneity, both cell lines (purple bars, Figure 2) and xenografts (black bars, Figure 2) are remarkably reproducible; we anticipate that adding additional xenograft models of ALL from distinct patients will mirror the heterogeneity of the patient from whom they were derived. It is also evident that overall, glucocorticoid-treated xenografts co-cluster with a group of 3 patients (B-ALL-37, -38, and -40), all of whom had BCP-ALL and a good early prednisolone response, with varied cytogenetics (hyperploidy, t(12;21), and normal respectively). At more relaxed clustering thresholds, the glucocorticoid-treated xenografts cluster with 4 other patients with BCP-ALL (B-ALL-24, -31, -33, and 43) and the cell lines.

We identified 5 clusters of gene sets with distinct expression profiles, each behaving differently in the 3 models of ALL. Cluster 1 demonstrated the markedly heterogeneous patterns seen in patient samples, with the xenograft samples showing a pattern similar to 8 of the patients; cluster 2 showed genesets that showed strong enrichment in the cell line study, and included a number of genesets associated with cell proliferation; cluster 3 did not show any striking differences across the three ALL models; cluster 4 showed genesets downregulated in both xenografts and cell lines compared to the patient samples, and included a number genesets associated with cell cycle progression, DNA/RNA replication and MYC; cluster 5 showed genesets strongly downregulated in the xenograft and cell line models, and included genesets associated with MYC and metabolic processes, particularly catabolism and energy production. In this limited comparison, it is clear that glucocorticoid-induced gene expression patterns seen in ALL are dependent on the experimental model, and that the patterns derived from the xenograft model show a greater similarity to patient-derived data than to cell lines.

A search of the TRANSFAC database v8.3 [41] of CoMoDis [42] identified GRE motifs (within 100 kb either side of the gene) in only 25 (14.5%) of the top 173 upregulated genes at the 8 hour timepoint in this study, and no GRE motifs were identified in the upregulated genes at 24 or 48 hours. This supports accumulating evidence that glucocorticoids exert long-range effects through very distal steroid receptor binding sites [43]. Analysis of significantly differentially expressed glucocorticoid-induced genes in an *in vitro *cell line study [13] revealed a similar number of early response genes after 6 hours of exposure (60 upregulated (t-stat > 10) and 27 downregulated (t-stat < -10)) but a significantly greater number of genes after 24 hours (593 upregulated (t-stat > 10) and 782 downregulated (t-stat < -10)). Interestingly, all but 2 of the genes upregulated at 6 hours remained significantly upregulated at 24 hours, and 17 of the downregulated genes at 6 hours remained downregulated at 24 hours. GRE motifs were identified in 15 (25.0%) of the top 60 upregulated genes at 6 hours, and 87 (14.6%) of the top 593 genes at 24 hours. The observed difference between the studies in gene expression at later timepoints is consistent with continuous rather than physiological glucocorticoid exposure. In addition, in the cell line study, the GR (NR3C1) undergoes highly significant early and sustained autoupregulation, which in the continuous presence of ligand drives downstream gene expression. In contrast, in the xenograft model minimal GR upregulation is seen at the early timepoint but no significant change in GR expression is seen at either of the later timepoints.

**Figure 3:**
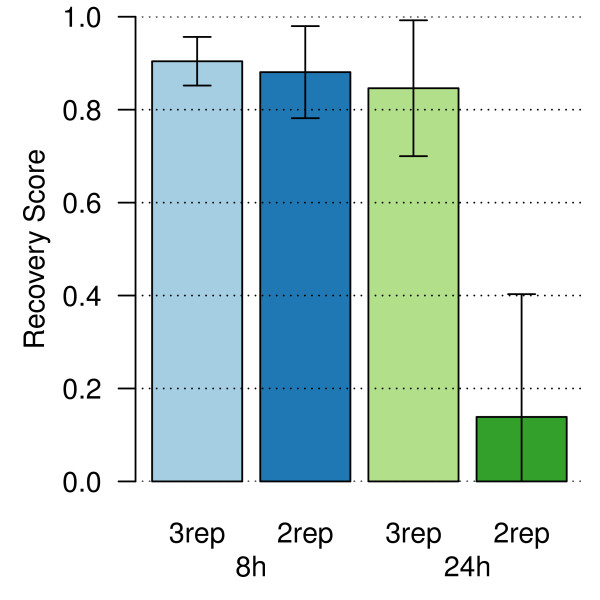
**Recovery scores at 8 hours and 24 hours when randomly selecting all combinations of 3 replicates (3rep) or 2 replicates (2rep) from the set of 4 biological replicates**. The Recovery Score represents the proportion of differentially expressed genes from all 4 replicates recovered when using fewer replicates.

Given the good statistical power observed in Figure 1A, we proceeded to determine whether we could use fewer replicates and still identify a majority of the differentially expressed genes. Replicate analysis (Figure 3) revealed that at the 8 hour dexamethasone-treated timepoint, a dataset with high signal and differential expression, using data from any 3 randomly chosen biological replicates instead of 4 resulted in excellent recovery scores of > 0.9. That is, on average, 90% of the differentially expressed genes identified from all 4 samples were also identified in any combination of 3 arrays. At 24 hours, a timepoint with less signal, the average recovery score was 0.85 with 3 replicates, but was more variable than at 8 hours. Using just 2 biological replicates recovered 88% of the list of differentially expressed genes at 8 hours, which dropped to 14% at 24 hours. This confirms that the 8 hour time point has the strongest signal, which is reproducible across different subsets of biological replicates. We recommend using a minimum of 3 biological replicates, since fewer replicates destabilized our ability to identify differentially expressed genes. This has important considerations for experimental design, and has significant implications on cost and animal numbers.

## Conclusions

We conclude that the NOD/SCID ALL xenograft mouse model provides biologically relevant insights into glucocorticoid-induced gene expression, in a consistent, reproducible and clinically relevant model system. We have demonstrated that the 8 hour timepoint provides the highest number of significantly differentially expressed genes, that time-matched controls are redundant and excellent recovery scores can be obtained with 3 replicates. We have thus established the optimal experimental design, with subsequent important implications for costs and animal numbers.

## Competing interests

The authors declare that they have no competing interests.

## Authors' contributions

VAB performed all experimental work and wrote the paper, VAB and MJC analyzed the data, TNT provided critical appraisal of the paper and WK and RBL designed the study. All authors read and approved the final manuscript.

## Supplementary Material

Additional file 1 **metaGSEA of genesets 8 hours after treatment with dexamethasone**. metaGSEA of top 100 up- and down-regulated genesets identified by Gene Set Enrichment Analysis (GSEA) 8 hours after treatment with dexamethasone. Click here for file

Additional file 2 **metaGSEA of genesets 24 hours after treatment with dexamethasone**. metaGSEA of top 100 up- and down-regulated genesets identified by Gene Set Enrichment Analysis (GSEA) 24 hours after treatment with dexamethasone. Click here for file

Additional file 3 **Annotated pGSEA comparing glucocorticoid-induced genesets in xenograft, cell line and patient datasets**. Hierarchical cluster by parametric Gene Set Enrichment Analysis (PGSEA) of the top 100 genesets with the greatest variance across three models (xenograft *in vivo*, cell line *in vitro*, patient *in vivo*) of glucocorticoid-induced gene expression in ALL, with annotation of the gene sets. Click here for file
