# Supplementary figures and images for: Evaluation of the NOD/SCID xenograft model for glucocorticoid-regulated gene expression in childhood B-cell precursor acute lymphoblastic leukemia
Source: BMC Genomics. 2011 Nov 17;12:565. doi: 10.1186/1471-2164-12-565 (PMC3228854; doi:10.1186/1471-2164-12-565)

# Leading Edge Similarities

Similarity (%)

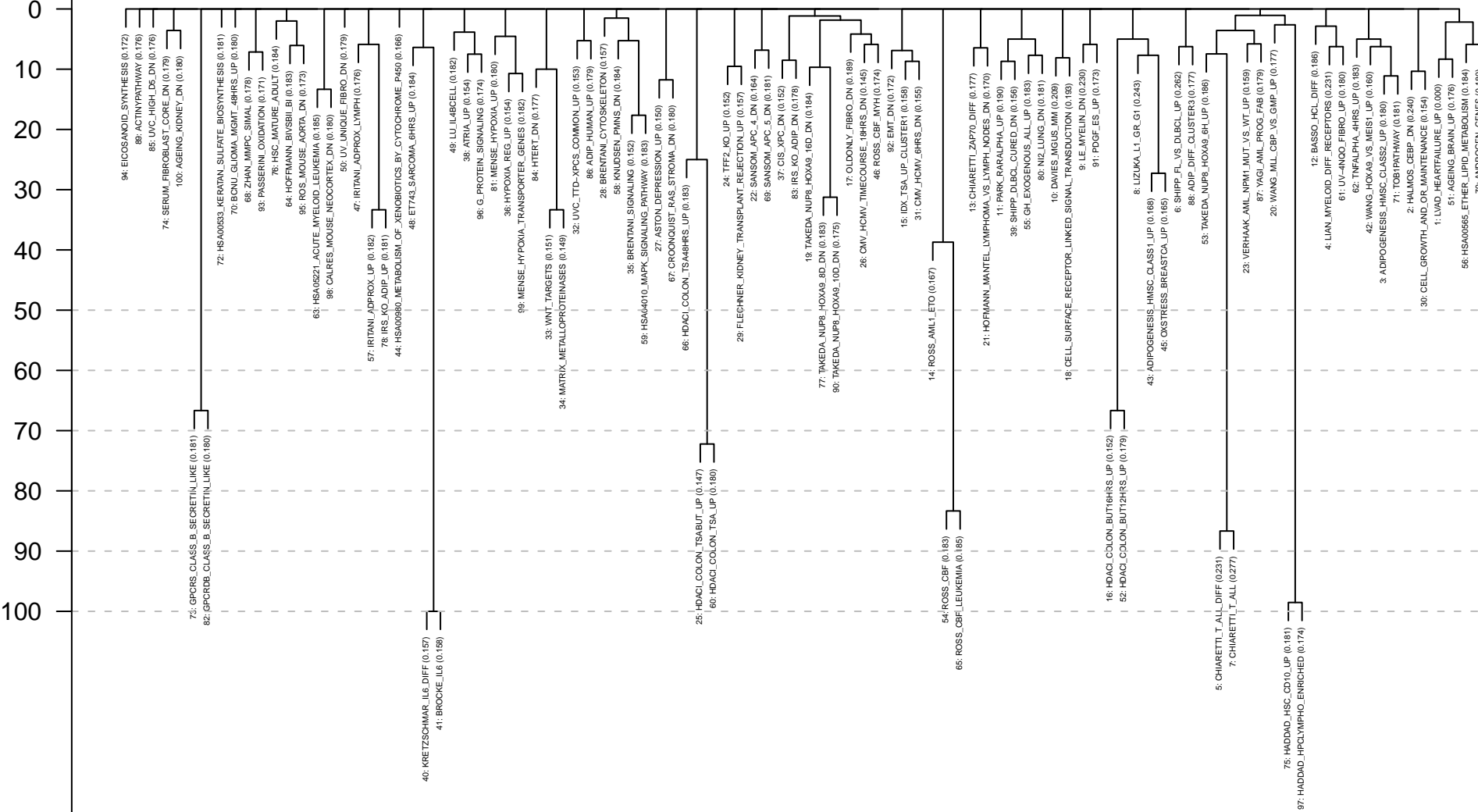

top 100 :: up

Leading Edge Similarities

Similarity (%)

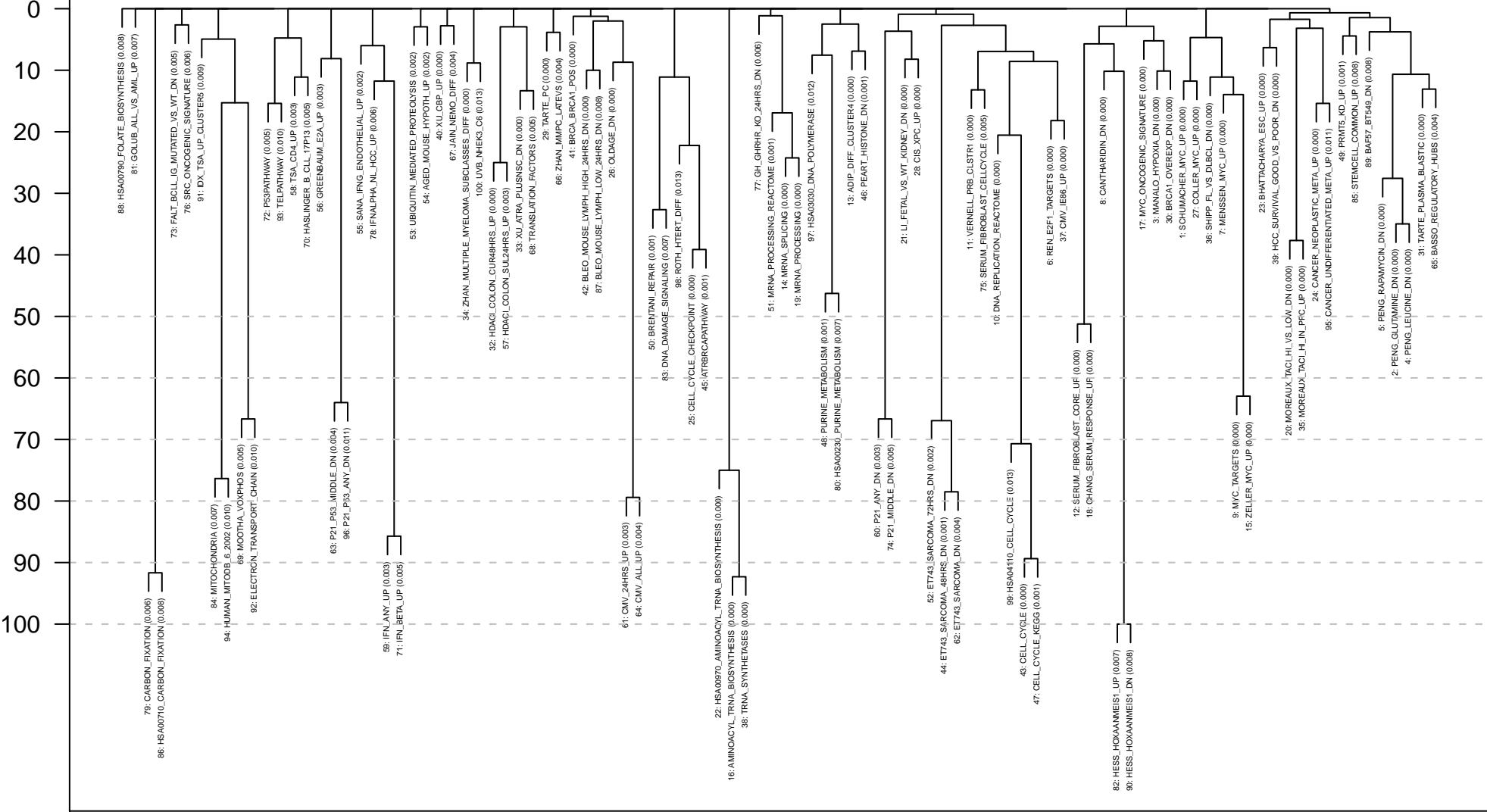

top 100 :: down

Supplement: Additional file 1 — metaGSEA of genesets 8 hours after treatment with dexamethasone. metaGSEA of top 100 up- and down-regulated genesets identified by Gene Set Enrichment Analysis (GSEA) 8 hours after treatment with dexamethasone. [file 1471-2164-12-565-S1.PDF]

# Leading Edge Similarities

Similarity (%)

0  
10  
20  
30  
40  
50  
60  
70  
80  
90  
100

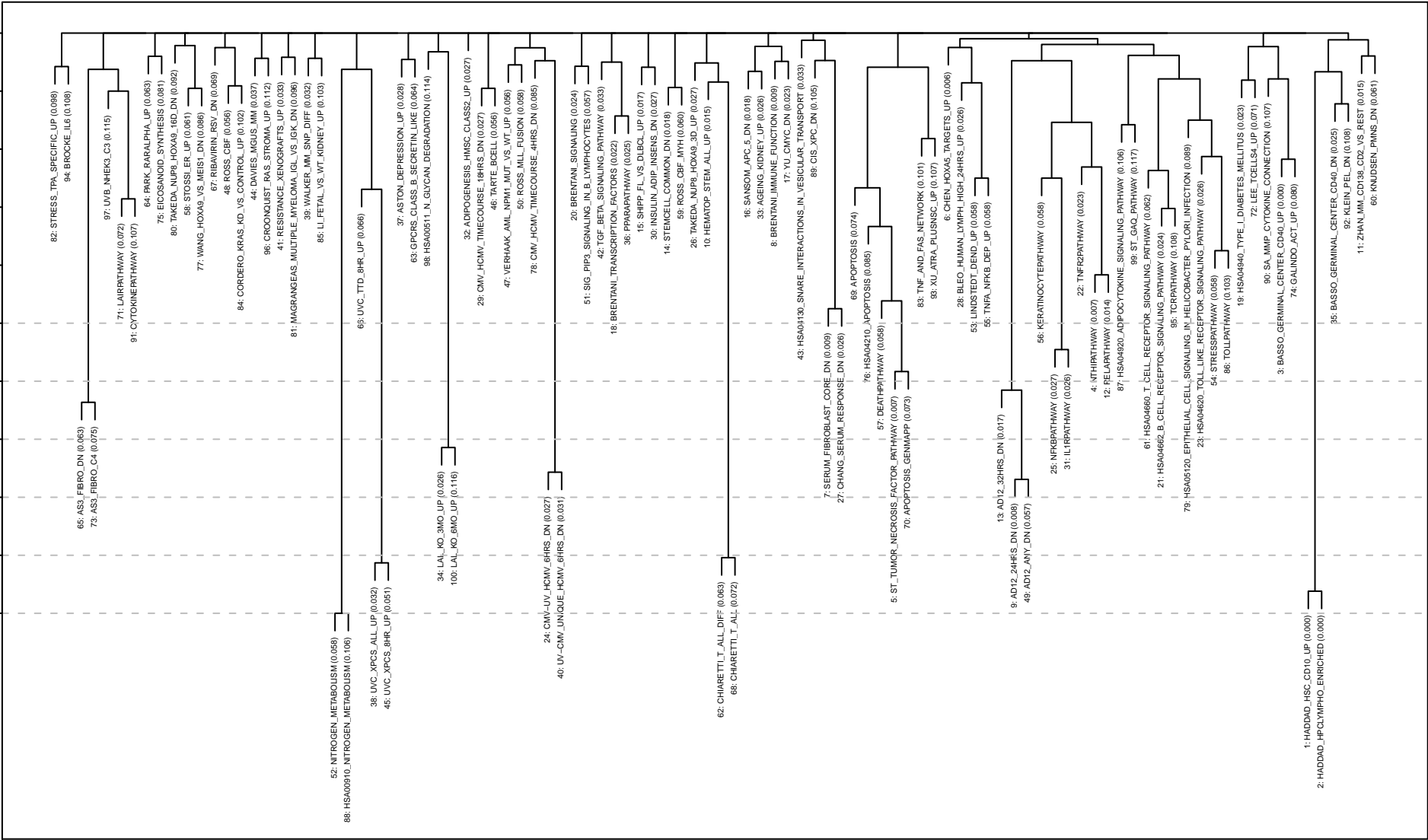

top 100 :: up

Leading Edge Similarities

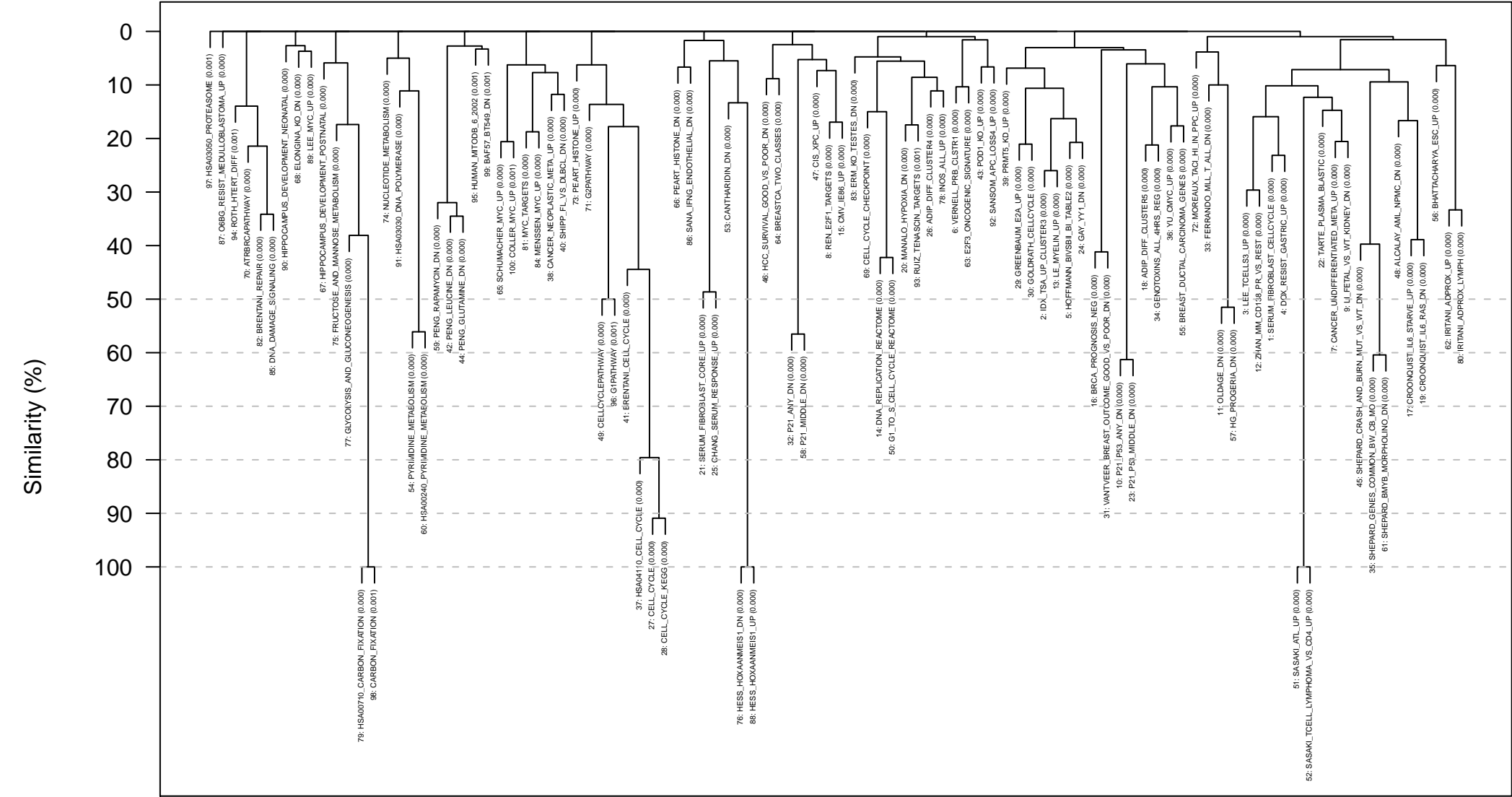

Supplement: Additional file 2 — metaGSEA of genesets 24 hours after treatment with dexamethasone. metaGSEA of top 100 up- and down-regulated genesets identified by Gene Set Enrichment Analysis (GSEA) 24 hours after treatment with dexamethasone. [file 1471-2164-12-565-S2.PDF]

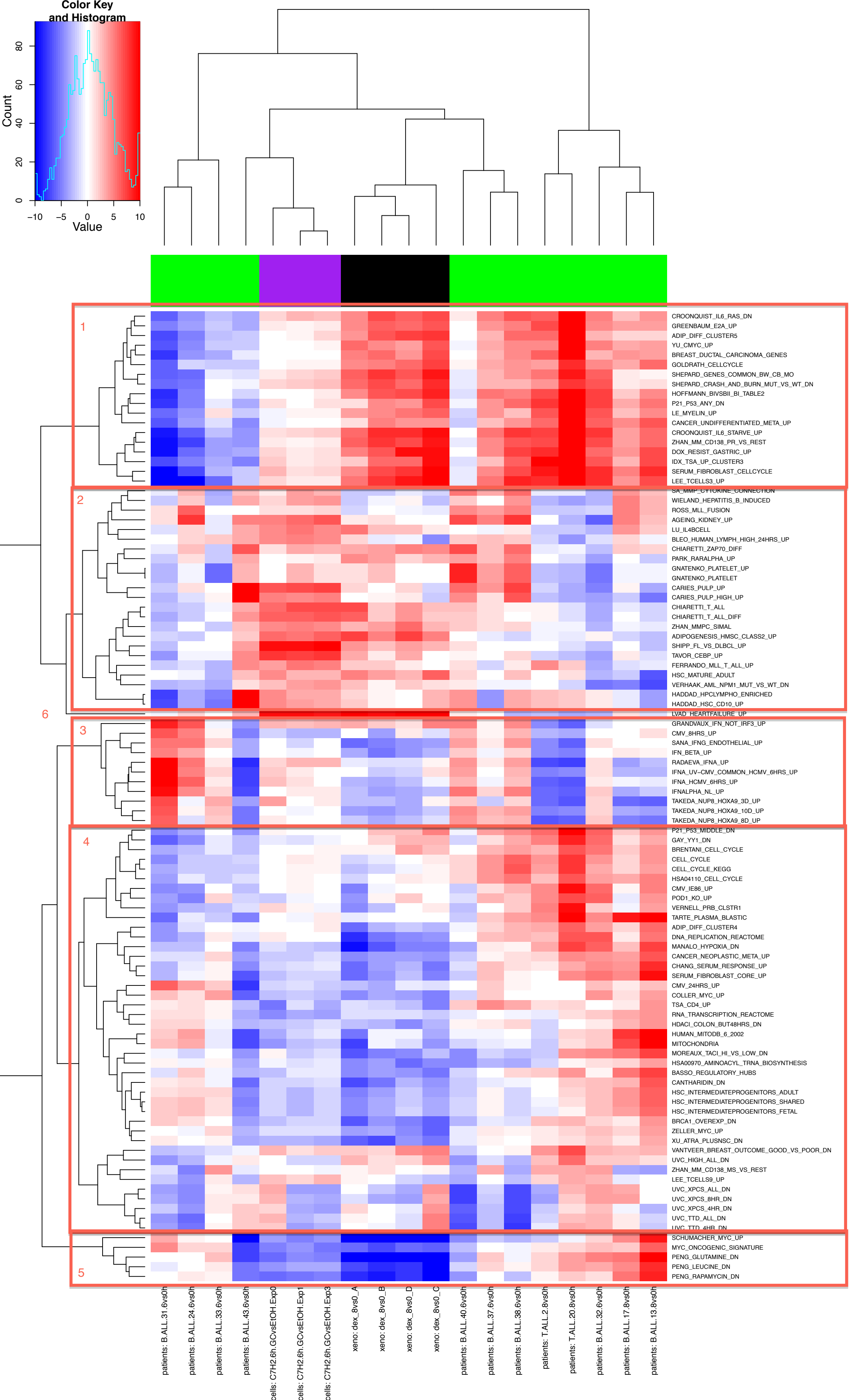

Supplement: Additional file 3 — Annotated pGSEA comparing glucocorticoid-induced genesets in xenograft, cell line and patient datasets. Hierarchical cluster by parametric Gene Set Enrichment Analysis (PGSEA) of the top 100 genesets with the greatest variance across three models (xenograft in vivo, cell line in vitro, patient in vivo) of glucocorticoid-induced gene expression in ALL, with annotation of the gene sets. [file 1471-2164-12-565-S3.PDF]
